# The Nutritional Impact of Milk Beverages in Reducing Nutrient Inadequacy among Children Aged One to Five Years in the Philippines: A Dietary Modelling Study

**DOI:** 10.3390/nu12113330

**Published:** 2020-10-29

**Authors:** Tsz-Ning Mak, Imelda Angeles-Agdeppa, Marie Tassy, Mario V. Capanzana, Elizabeth A. Offord

**Affiliations:** 1 Nestlé Institute of Health Sciences, Nestlé Research, Route du Jorat 57, 1000 Lausanne, Switzerland; Marie.Tassy@rd.nestle.com (M.T.); elizabeth.offord-cavin@rdls.nestle.com (E.A.O.); 2 Department of Science and Technology-Food and Nutrition Research Institute, Taguig City 1631, Philippines; iangelesagdeppa@yahoo.com.ph (I.A.-A.); mar_v_c@yahoo.com (M.V.C.)

**Keywords:** dietary modelling, nutrient inadequacy, dairy, milk, recommendations

## Abstract

Around half of Filipino children are not consuming any dairy products on a given day, which has shown to be associated with increased risk of inadequate nutrient intakes. The current study applies dietary modelling to assess the nutritional impact of meeting dairy recommendations in reducing nutrient inadequacy in children aged one to five years in the Philippines. Dietary intake data of Filipino children aged one to five years (*n* = 3864) were analyzed from the 8th National Nutrition Survey 2013. Children who did not meet national dairy recommendations were identified. Two scenarios were applied, based on two types of commonly consumed milk products by the survey participants. In scenario one, one serving of powdered milk was added to the diet of these children. In scenario two, one serving of a young children milk (YCM) or preschool children milk (PCM) was added to the diet of children aged one to two years and three to five years, respectively. Mean nutrient intakes and percentages of children with inadequate intakes were estimated before and after applying modelling scenarios. Scenario one demonstrated improvement in calcium, phosphorus, sodium, vitamin A and riboflavin intakes, while in scenario two, further improvement of intakes of a wider range of nutrients including iron, selenium, zinc, magnesium, potassium, vitamins C, D, E, thiamin, niacin, vitamins B6, and B12 was observed. In both scenarios, if all children would meet their dairy recommendations, theoretical reductions in population nutrient inadequacy would be observed for all micronutrients, for example, only 20% of children aged one to two years would be inadequate in vitamin A instead of the current 60%, iron inadequacy would see a 5% reduction, and approximately 10% reduction for calcium and 20% reduction for folate. The present study is the first to apply dietary modelling to assess the theoretical impact of meeting dairy recommendations on nutrient inadequacy in children in the Philippines. Dairy consumption should be encouraged as part of the strategy to reduce nutrient inadequacies. Calcium, iron, vitamins D, E, and folate are of concern in the Philippines as the level of inadequacies are extremely high in early years, YCM and PCM can help increase the intake of these nutrients.

## 1. Introduction

Suboptimal diet quality and nutrient inadequacy are common among children in the Philippines [1,2]. Previous studies have found that mean energy intakes among these children are approximately 30% below the Philippines recommended energy intake, and those who consume no milk or dairy have energy intake close to 50% below the recommendation [1]. The dietary pattern of Filipino children is mainly comprised of refined rice and low-nutrient-dense foods (e.g., cookies and other sugary foods), and limited quantity of major food groups such as fruit, vegetables, meat and dairy [2].

Around 50% of Filipino children are not consuming any dairy products on a given day [1], despite the national recommendation of one glass of milk or milk product per day for children aged one to six years [3]. Milk and dairy products are key sources of nutrients such as energy, protein, and calcium for normal growth and development in young and preschool children. Recent evidence suggests that Filipino children who do not consume any milk or dairy products have a very low likelihood of meeting the recommendations for protein, total fat, calcium, iron, zinc, B vitamins, and vitamins C, D, E [1].

Increasing the consumption of milk products among children who do not typically consume any dairy, may help to improve energy intakes and reduce nutrient inadequacy. To test this hypothesis, dietary modelling can be a useful way to assess the theoretical nutritional impact of increasing dairy consumption at a population level. Dietary modelling, also referred to as food pattern modelling, is a statistical technique that has been increasingly applied to inform development of dietary guidelines in parts of the world, including the United States, Australia, United Kingdom, and Ireland [4,5,6,7]. The 2020 Dietary Guidelines for Americans Advisory Committee defined it as a method to understand how changes to the amounts or types of foods and beverages in a dietary pattern might impact meeting nutrient needs [6]. The process involves the modification of elements within the typical diet of a population, such as food groups, food group amounts, inclusion and exclusion of certain foods, nutrient profiles of foods, diet goals or constraints, on the theoretical impact of nutrient intakes across the said population [6].

The Food Safety Authority of Ireland recently applied dietary modelling to develop food-based dietary guidelines for children 1–5 years. Irish children are at risk of inadequate iron intakes (aged one to three years) and vitamin D intakes (aged one to five years) [7]. Dietary modelling showed that an average consumption of 442 mL/day follow-up formula fortified with iron, 30 g of iron-fortified (12 mg/100 g) cereal five days a week and red meat (30 g) three days a week would meet adequate iron intakes among one-year old children. For children aged 1.5 to 3 years, consuming 330 mL/day of young child formula fortified with iron (1.2 mg/100 mL) would be enough to meet iron needs [7]. As for vitamin D, food fortification in milk and cereal as well as supplementation would be needed. Though systematic fortification of foods with vitamin D is not common in Ireland, except for fortified follow-up formula and young child formula [7].

One US dietary modelling study found that the prevalence of inadequate calcium, potassium and magnesium intakes across the US populations (aged two years and over) would theoretically reduce if additional servings of dairy foods were consumed [8]. Another study showed that when modelling dairy consumption at recommended amounts, essentially all Americans would meet estimated average requirement (EAR) of calcium intake, and vitamin A and magnesium inadequacy would significantly reduce [9]. One example from China demonstrated that when adding amounts of formulated milk or cow’s milk to help children three to eight years reach their recommended dairy consumption, their nutrient intakes would improve while micronutrient inadequacy such as vitamin A, C, iron, zinc would reduce considerably [10].

While previous studies in the Philippines support that dairy consumers have higher nutrient intakes and lower nutrient inadequacy than non-dairy consumers, the theoretical impact of meeting dairy recommendations on nutrient intakes at the population level is not known. The current study therefore aims to apply dietary modelling to assess the nutritional impact of adding a serving of dairy (based on two types of commonly consumed milk beverages) to the diets of children (aged one to five years) who do not meet their dairy recommendations, to estimate the impact of fulfilling dairy recommendations on nutrient adequacy.

## 2. Materials and Methods

### 2.1. Study Population

The 2013 Filipino National Nutrition Survey (NNS) data on children aged one to five years (*n* = 3902) were examined. The 2013 NNS was a cross-sectional, nationwide representative survey of the Filipino population on health and nutrition, providing quantitative information on food intake by households and individuals. The Food Nutrition Research Institute (FNRI) Ethics Committee accepted the survey protocol and resources for data collection. Prior to participation with the registry number FIERC-2013-001, all households surveyed issued informed consent [11].

### 2.2. Data Collection

During household visits, anthropometric measurements, including weight and height, and demographic details, such as wealth status, housing status, were collected. The 24 h dietary recalls were performed face-to-face by professional dietitians with a parent or caregiver. All food and beverages consumed by the child on the previous day were registered. Around two days after the first recall, fifty percent of randomly selected households had a second 24 h recall. The amount of food and beverages consumed was measured using household scales, such as cups, teaspoons, size or number of bits, and converted to grams using a serving-to-weight list of popular foods compiled by FNRI. Quality tests have been performed to ensure that the recorded foods and drinks and amounts have been correctly entered. Further detail on the processing of dietary data can be found in the previous publication [2]. All recorded foods and beverages were classified into nine main food groups and subgroups. Food and beverages consumed have been translated to energy and nutrient intakes using the Philippines food composition tables (PFCT) [12]. The PFCT reflects, to some extent, mandatory fortification of certain foods including cooking oil (with vitamin A), wheat flour (with vitamin A and iron) and rice (with iron). Information on dietary supplement use was not collected in 2013 NNS, and therefore not included in the daily nutrient intakes estimations except for vitamin A. In the Philippines, children between the age 1 to 5 years receive high-dose vitamin A supplementation twice per year [13]. While evidence suggests the coverage of the supplementation program is around 83% [14], the current study accounted for the potential impact of vitamin A supplementation by assuming every child received a dosage of 200,000 IU RE (60 mg) vitamin A capsule every 6 months, which translates to approximately 330 µg RE of vitamin A per day.

### 2.3. Data Processing

For each participant, the total daily intake of 27 nutrients was calculated, including energy, macronutrients: carbohydrates, total sugar, fiber, protein, total fat, saturated fat, monounsaturated fat, polyunsaturated fat, minerals: calcium, iron, magnesium, phosphate, potassium, selenium, sodium, zinc, vitamins: thiamine, riboflavin, niacin, vitamin B6, folate, vitamin B12, vitamin C, vitamin D, and vitamin E. For children who had a second recall day, the average intake of each nutrient over the two recall days was considered in the subsequent analyses. The distribution of energy intake was assessed and intakes that were three standard deviations above and below (±3 SD) the mean per age group were considered implausible and therefore removed from the analyses. Only non-breastfed children were included in the analyses to respect the milk code in the Philippines. A final sample of 3864 children was retained for analysis.

All food and beverages consumed by participants were assigned to 10 major food groups and subgroups (as described previously) [2]. The key food group “Milk and milk products” was further sub-categorized into infant formula (powdered), toddler/preschooler formula (powdered), milk (liquid and powdered), cheese and yoghurt. The number of servings of milk and milk products consumed by each child was determined by applying the Philippines government’s daily nutritional guide pyramid serving definitions [3]. A serving of milk or milk product is defined as 180 g of liquid milk (full, semi-skimmed low-fat, reconstituted powdered, colored, fortified milk, butter milk), or 160 g of condensed or liquid evaporated milk, or 28 g of powdered milk, or 90 g of yoghurt, or 20 g of cheese. Children who consumed no milk or less than one serving of milk and milk products were considered as not meeting their dairy recommendations.

### 2.4. Modelling Scenarios

Among children not meeting dairy recommendations, two dietary modelling scenarios were applied. In scenario one, one serving (180 g) of powdered milk (the most common milk reported in the survey was reconstituted powdered milk, coded as “Milk, powder, filled, instant”) consumed by 28% of children aged one to five years was added to the diet of these children. In scenario two, one serving (180 g) of a popular young children milk (YCM) in the Philippines was added to the diet of children aged one to two years, and one serving of a popular preschool children milk (PCM) was added to the diet of children aged three to five years. The reasons for applying two scenarios (milk and YCM/PCM) were because, firstly, these are the most commonly consumed dairy options for this age group based on the national survey, and secondly, these milks contribute differently to nutrient intakes among children in the Philippines, due to different fortification levels. Powdered milk, also referred to as filled milk, is a milk reconstituted with vegetable oils, aimed for the use of the whole family and highly fortified with nutrients such as calcium and vitamin A. YCM and PCM are fortified with a wider range of nutrients such as fiber, polyunsaturated fatty acids, vitamins and minerals, tailored for the nutrient requirements of children aged one to two years and aged three to five years, respectively. The composition of the milk beverages used in the modelling scenarios can be found in Table S1. Total daily nutrient intakes and nutrient inadequacy before and after applying the scenarios were calculated.

### 2.5. Statistical Analysis

Data were analyzed using R version x64.3.6.1 and R-Studio version 1.2.1335. Analyses were split by the following age groups: one to two years (12 to 35.9 months), three to four years (36 to 59.9 months), and five years (60 to 71.9 months). Descriptive statistics of the sample (gender, age group, dwelling location, wealth status, and BMI z-scores) and whether children met or did not meet dairy recommendations were summarized using chi-squared tests. Mean nutrient intakes were calculated per age group before and after applying scenarios one and two. Due to the skewed distribution of nutrient intakes, the non-parametric Wilcoxon signed-rank test was used to test the difference in nutrient intakes between scenario one and scenario two. The Philippines dietary reference intakes (PDRI) table [15] was used to determine the reference values for nutrient intakes. The acceptable macronutrient distribution ranges (AMDR) were used to evaluate carbohydrates and total fat, as a percentage of energy (%E). The proportions of inadequate macronutrient intakes were classified as %E less than the AMDR lower range. The estimated average requirements (EAR) cut-off method was used to establish the percentage of children with inadequate intakes, except for iron, as iron intake and requirements are highly skewed in young children. The probability approach was used to estimate the prevalence of iron inadequacy, based on the Institute of Medicine iron probability of inadequacy estimation [16]. The probability was adapted based on an assumption of 8% bioavailability of iron from food [17] to reflect the typical Filipino diet low in haem iron from meat sources. For nutrients that did not have an established EAR value, e.g., vitamin D and vitamin E, adequate intake (AI) values were used. The proportion of children with nutrient intakes below the reference values were calculated to establish prevalence of inadequate intakes before and after applying the scenarios. The proportions of children with inadequate intakes before and after applying the scenarios across the full sample (all children regardless of meeting dairy recommendations or not) were also calculated. Regarding the assumption of every child receiving vitamin A supplementation of approximately 330 µg RE of vitamin A per day, the percentage of children above the upper limit for vitamin A for each scenario were also estimated (Supplementary Table S2).

## 3. Results

### 3.1. Sample Characteristics

Two-thirds of children (68%; *n* = 2642) did not meet the dairy recommendations of one serving per day (Table 1). The proportion of children not meeting dairy recommendations were higher in the older age groups; at age five years, only 18% of children met the recommendations. Fewer children from urban areas met the dairy recommendations compared to those from rural areas. Interestingly, for wealth status, the highest percentage of children who met the dairy recommendations came from the wealthiest quintile, but the highest percentage of children who did not meet the recommendation were not those from the lowest wealth quintile (poorest). Those from the middle wealth quintile had the highest proportion of children not meeting the dairy recommendations, followed by the poor quintile. A similar trend was also observed for BMI z-score, whereby the highest proportion of those not meeting dairy recommendations were not children who were at risk of wasting but those with normal BMI Z-scores (0 to −2).

### 3.2. Nutrient Intakes of Children Who Did Not Meet Their Dairy Recommendations (Baseline Intakes)

Children who did not consume the recommended one serving of dairy per day had mean nutrient intakes far lower than the reference values (EAR or AI). Among children aged one to two years, only mean intakes of protein, sodium, selenium, niacin, vitamin B12, and vitamin C were at or above the reference intakes (Table 2). Mean intakes of energy, total fat, fiber, iron, vitamin D and vitamin E were particularly low. Mean energy intake was 556 kcal (SD ± 337 kcal) per day when the estimated energy requirement for this age group was 920 kcal (girls) and 1000 kcal (boys). For iron, mean intake was 3 mg (SD ± 2.6 mg) per day (EARs: 6.4 mg for boys and 7 mg for girls), mean calcium intake 128 mg (SD ± 105 mg) was less than half of the EAR for calcium (440 mg) and mean vitamin D was 1 μg (SD ± 2 μg) while adequate intake was 5 μg. Similar trends could be seen for older children (three years and above), mean intakes of energy, total fat, fiber, calcium, iron, zinc, folate, vitamins D and E were far below the recommendations (Table 3 and Table 4).

### 3.3. Comparison of Nutrient Intakes before and after Modelling

As expected, after adding a serving of milk (scenario one) or YCM/PCM (scenario two) to the diets of children who did not meet their dairy recommendation, their theoretical nutrient intakes increased and were brought closer to the EAR or AI of the nutrients. In both scenarios, energy and macronutrient intakes improved across all ages. In children aged one to two years, one serving of powdered milk provided higher intakes of energy, protein, fat intakes, whilst YCM provided higher carbohydrates and fiber intakes (Table 2). The same trend was observed in children aged five years (Table 4). Among children aged three to four years, powdered milk provided higher macronutrient intakes compared to baseline, whilst PCM provided higher fiber (Table 3).

In scenario one, compared to baseline, adding a serving of powdered milk to the diets of children one to two years increased sodium, calcium, phosphorus, vitamin A, riboflavin and vitamin C intakes and moderately improved potassium, zinc, thiamin intakes but provided little or no improvement in iron, magnesium, selenium, B6, B12, folate, vitamin D and vitamin E intakes. In children aged three to four years, adding a serving of powdered milk improved the intakes of all nutrients to some extent. The biggest improvement was seen in calcium, phosphorus and vitamin A. In children aged five years, similar trends were observed as for children aged one to two years, whereby adding a serving of powdered milk improved intakes of calcium, phosphorus, vitamin A, riboflavin and vitamin C.

In scenario two, compared to baseline, adding a serving of YCM or PCM to the diets of children improved all micronutrient intakes. The biggest increase in intakes was seen in iron, selenium, zinc, magnesium, potassium, vitamin C, vitamin D, vitamin E, thiamin, niacin, vitamins B6 and B12, and folate, across all three age groups.

While both scenarios would theoretically improve the nutrient intakes of children, meeting the dairy recommendation with a serving of powdered milk would provide higher intakes of sodium, calcium, phosphorus, vitamin A and riboflavin compared to YCM or PCM. On the other hand, a serving of YCM or PCM would significantly increase the intakes of iron, selenium, zinc, magnesium, potassium, vitamins C, D and E, thiamin, niacin, vitamins B6 and B12 compared to powdered milk (all *p* < 0.001).

### 3.4. Comparison of Nutrient Inadequacy before and after Modelling

Among the no/low milk consumers aged 1 to 2 years, scenario one helped to reduce inadequacy of calcium, phosphorus, vitamin A and riboflavin compared to baseline (Table 5). In particular, in this scenario, the percentage of no/low milk consumers with riboflavin inadequacy would be zero. Scenario two would reduce inadequacy across a wide range of nutrients, including fiber, magnesium, selenium, thiamin, niacin, vitamins B6, B12, folate, vitamin C, D and E. In this scenario, all children would meet the EAR for vitamin C. Similar patterns can be observed for the older age groups (Table 6 and Table 7).

The nutritional impact of applying scenarios one and two to the total sample of the three age groups (including children who did and did not meet dairy recommendations) are shown in Table 5, Table 6 and Table 7. These scenarios demonstrated that if all children were to meet their dairy recommendation with either type of milk beverage, the proportion of children across the Philippines with inadequate intakes would be reduced.

If all children aged three to four years were to consume one serving of powdered milk per day, the level of nutrient inadequacy across the population would be reduced for protein (from 22% to 7%), total fat (from 45% to 22%), sodium (from 26% to 17%), calcium (from 84% to 62%), phosphorus (from 48% to 16%), riboflavin (from 52% to 3%) and vitamin A (from 56% to 13%) (Table 6). No difference in inadequate levels was seen in other nutrients.

On the other hand, if all children aged 3 to 4 years were to consume one serving of PCM per day, the population inadequacy would be reduced for iron (81% to 71%), magnesium (44% to 29%), selenium (8% to 4%), zinc (60% to 34%), vitamin A (56% to 36%), thiamin (54% to 29%), riboflavin (52% to 19%), niacin (25% to 11%), vitamin B6 (48% to 21%), vitamin B12 (44% to 25%), folate (73% to 56%), vitamin C (68% to 14%), and vitamin E (94% to 87%). A small decrease in vitamin D (from 93% to 90%) and fiber (from 94% to 91%) inadequacies was seen in this scenario. Similar observations were found for other age groups.

## 4. Discussion

Using dietary modelling, the current study applied theoretical scenarios to add a serving of milk products to the diets of children who did not meet their dairy recommendations to assess the impact of dairy on nutrient intakes among children of one to five years in the Philippines. Overall improvement of macro- and micronutrient intakes was observed, but also the types of milk products contributed differently to nutrient intakes in children. Adding a serving of powdered milk would see the most improvement in calcium, phosphorus, sodium, vitamin A and riboflavin intakes. While adding a serving of YCM or PCM would improve, in addition to those found in powered milk, a range of nutrient intakes including iron, magnesium, potassium, selenium, zinc, thiamin, niacin, vitamins B6, B12, C, D and E. Most of these nutrients are highly relevant in the context of the Philippines, as previous studies have found that inadequacies were particularly high in calcium, iron, zinc, phosphorus, vitamins A, D, E and folate among young and preschool children in the Philippines [1,2]. Adding these milk products to the diets of children can improve the intakes of nutrients that are essential for normal growth and development in young children, including normal function of the immune system (e.g., folate, iron, selenium, zinc, vitamins A, B6, B12, C, and D), normal cognitive function (e.g., iron, zinc), and normal bone growth and development (e.g., calcium, vitamin D, phosphorus, and protein) [18,19].

### 4.1. Meeting Dairy Recommendations and Reducing Nutrient Inadequacy

The current study also estimated the theoretical reduction in population nutrient inadequacy if all children were to follow the government’s dairy recommendation of one serving of milk and milk products. For those who did not meet the dairy recommendation, having a serving of either powdered milk or YCM/PCM would improve the level of nutrient inadequacy for the 23 nutrients tested in this study. For instance, with a serving of powdered milk, the estimated population inadequacy in vitamin A would be 20% instead of the current 60%. Likewise, for riboflavin, population inadequacy would be 5% instead of 48%. Powdered milk has little impact on reducing population micronutrient inadequacy beyond sodium, calcium, phosphorus, vitamin A and riboflavin. On the other hand, among those who did not meet dairy recommendation, if they had a serving of YCM or PCM per day, population nutrient inadequacy would be reduced for a wide range of micronutrients, including iron, magnesium, zinc, and all vitamins. For example, in children aged 5 years, population inadequacy for iron would reduce from 79% to 65%, for zinc from 56% to 26%, for vitamin C from 69% to 10%, and for vitamin D, probably the most difficult nutrient to achieve adequacy, from 93% to 88%, with one serving of PCM.

The current study has identified an opportunity to improve nutrient intakes and adequacy with milk products in children in the Philippines through the application of dietary modelling, the first of its kind in this geography. While this study cannot directly compare to previous studies in the Philippines, some similarities and differences can be highlighted when compared to modelling studies from the US, China, and Ireland on toddlers and preschool children. A study in China observed positive nutritional impact of adding a formulated milk powder for children three to eight years, whereby reductions in vitamin C, thiamin, vitamin A, iron, zinc and potassium inadequacy were seen [10]. In Ireland, while calcium inadequacy is not a public health issue for children due to the large volume of milk typically consumed, it has been observed that vitamin D and iron adequacy are difficult to achieve among children aged one to four years. The modelling study found that the best dietary strategy to meet vitamin D and iron adequacy in children aged one and two years was to either fortify all cow’s milk with vitamin D at 2 µg/100 mL and iron at 1.2 mg/100 mL and/or to replace all cow’s milk with growing up milks that are typically fortified with vitamin D [20]. A US study based on NHANES (National Health and Nutrition Examination Survey) data found that when dairy intakes were modelled at recommended levels, children were no longer inadequate in calcium and magnesium. However, for vitamin D, 92% of children two to three years and 98% of children aged four to eight years would still be far below the EAR [9]. Such small reductions in inadequacy were also observed in the current study for vitamin D, confirming vitamin D population adequacy is extremely difficult to meet across different geographies. Vitamin D fortification in a range of commonly consumed products, as well as an increase in public health education to encourage higher intakes are important strategies to improve adequacy.

It is important to highlight other strategies that may help improve nutrient intakes and address nutrient inadequacy, some may be more cost-effective than others, while some may be of higher efficacy. In the Philippines, the Food Fortification Act of 2000 mandates food fortification to increase availability of vitamin A and iron to the nation through staple foods [21]. For example, cooking oil is fortified with vitamin A, wheat flour with vitamin A and iron, and rice with iron. Rice is a good example of where mandatory fortification works—it is the top food source of iron among children aged two years and above due to the large quantity of rice consumed daily [2]. In children younger than two years old, iron-fortified infant formula or young children milk beverages are the top sources of iron as the diets of these children are still predominately milk-based [2]. There are challenges, however, with large-scale food fortification programs, such as compliance [22,23]. The Department of Health (DoH) also encourages voluntary fortification of all processed food products in the Philippines, as a strategy to improve nutrient intakes across the population. Products that comply with the criteria set out by the DoH can carry a seal signifying approval of the Sangkap Pinoy Seal Program [23]. Voluntary fortification may help to address other nutrient inadequacies that are not covered by the mandatory fortification program, and to provide a level playing field for the food industry to share public health nutrition responsibilities.

Children between six months to five years receive high dose vitamin A supplementation twice yearly. Despite this, vitamin A deficiency (VAD) is one of the top public health problems in the Philippines, with 19.6% of children aged one to five years having VAD in 2013 [24]. As shown in Table S2 in Supplementary Materials, assuming a dosage of 60 mg RE of vitamin A every six months, each child would theoretically receive a daily equivalent of 330 µg RE of vitamin A, just below the daily recommended nutrient intake of 400 µg RE. In the current study, powdered milk or YCM/PCM provide approximately 200 or 100-140 µg RE per serving respectively. Thus, it is important to be aware of the upper limit of vitamin A (600 µg RE for 1 to 3 years; 900 µg RE for 4 to 5 years) when combining supplementation with several fortified food sources. Nevertheless, VAD is still an issue in the Philippines.

Children, particularly of this age group, should be encouraged to form healthy eating habits that will continue throughout childhood and into adulthood. Following the Philippines nutritional guide pyramid would enable children to have a balanced and diverse dietary pattern, incorporating a range of food groups and nutrients to fulfill their daily requirements. However, evidence suggests that dietary patterns of young and pre-school children in the Philippines are far from ideal. In children as young as one year old, 25% of children eat cookies, 20% consume sugar sweetened beverages (SSBs), 19% consume table sugar, and 9% cake. These percentages increase as children get older. By age three years, SSBs are the fourth top consumed food/beverage [2]. Unfortunately, given its high consumption, SSBs are often used as a vehicle for fortification of micronutrients [25], which may somewhat encourage consumption. With the country going through nutritional transition with increased prevalence of overweight and obesity, from the public health standpoint, it is important to focus on consuming nutrient dense foods to meet dietary needs.

### 4.2. Dairy Recommendations

Half of the children aged one to two years did not meet the dairy recommendations of one serving per day. This increases to three-quarters of those aged three to four years, and by age five, four in five did not meet their dairy recommendations. The decreasing trend in dairy consumption, particularly for milk, is consistent with findings from other parts of the world [9,26]. One interesting finding from the current study is that the highest proportion of children not meeting dairy recommendations were not those from the poorest households, but the middle-income households, suggesting that wealth may not be a barrier to low dairy consumption. Perhaps food choices, accessibility and availability differ between middle-income areas versus high- or low-income areas. Moreover, it was children with normal weight-for-height, rather than those at risk of wasting, that were not consuming enough dairy. It may be possible that dairy products are being displaced by other food and beverages in the diet, for example, increased sweetened beverage intake is linked to reduced milk consumption and calcium intake [15]. A previous study observed that 20% of children aged one-year consumed sugar sweetened beverages on a given day, this increased to 33% among children aged two years and 43% among aged three to four years. Other low-nutrient-dense foods such as cookies, cakes and candy were amongst the top 20 most consumed foods by Filipino children [2]. These foods are unlikely to contribute to micronutrient intakes in these children. In contrast, milk products including toddler milk are the top sources of thiamine, riboflavin, vitamin A, vitamin C, calcium, iron and zinc at age one year. In older children, given their low milk consumption, rice, fish, vegetables, sausages and luncheon meat become key sources of these nutrients [2]. However, because the quantity typically consumed is small (except for rice), the overall contribution of these food sources to total micronutrient intakes remains low, giving rise to high prevalence of population inadequacy. While national strategies such as supplementation and large-scale food fortification can help improve nutrient intakes at the population level with minimal cost and efforts, there are ways to improve the nutritional intakes of children at the household level. Educating and encouraging parents to choose nutrient-dense natural or fortified foods, increasing diet diversity and consumption of all key food groups can help their children increase micronutrient intakes [27]. While financial constraints may be a determinant of dietary habits and intakes in some Filipino households, it is important to keep in mind that some of the widely available foods in the Philippines are also highly nutritious and affordable, such as mung beans, moringa, fish and bananas. The current study showed that, surprisingly, those from the middle wealth bracket had the lowest consumption of milk products, suggesting their preference for other foods.

The current Philippines nutritional guide pyramid recommends Filipino children aged one to six years should consume one glass of milk per day [3]. Our modelling study finds that even after adding a serving of milk product to the diets of children to meet this recommendation, mean calcium intakes across all ages, while increased significantly, were still below the estimated average requirements, and the majority of children still inadequate. Therefore, a single glass of milk is unlikely to meet the calcium needs of children at age one to five years. Given that yoghurt and cheese are not commonly consumed in this population of children, perhaps increasing the recommendation to two glasses or more of milk and milk products per day could be considered to improve nutrient intakes. Such recommendation would be in line with daily dairy recommendations in other Asian countries: Japan two servings, Malaysia one to three servings, Thailand one to two servings, China 300 mL, and 500 mL in India and Nepal [19].

### 4.3. Strengths and Limitations

The current study, to our knowledge, is the first in the Philippines to use dietary modelling to test the impact of meeting dairy recommendations to reduce nutrient inadequacy. Dietary modelling is a very useful and scientifically recognized technique to assess the impact of theoretical changes to food or beverage consumption in relation to nutrient intakes. While the modelling is theoretical, the study shows the potential of reducing the nutrient gap for most nutrients at the population level by simply adding a serving of milk product to the diets of children aged one to five years. The fact that the dietary modelling was performed using the large sample of national survey data enabled the habitual dairy consumption patterns to be respected, and that the results would be representative across the children in the Philippines.

The results of dietary modelling should be interpreted with caution. While the scenarios were realistic, it was a hypothetical statistical exercise. Dietary modelling could not predict the behavior of individuals in reality and the resulting reduction in inadequacy was likely to be the “best case scenario” if all children would comply to the recommendations. The current study also did not investigate the impact of dietary modelling on excessive nutrient intakes due to a large proportion of the study population being highly inadequate in micronutrients. Furthermore, given that the energy and nutrient intakes were so low among these children, underreporting in the 24 h recall may be possible. Parents may not fully and accurately recall all food and beverages, as well as portion sizes consumed by their children, particularly if they spent part of the day being cared for by others. Lastly, total nutrient intakes could be underestimated due to supplement use not being captured in the 2013 NNS, and the Philippines food composition table may not fully reflect the level of mandatory or voluntary fortifications in all products.

## 5. Conclusions

Through dietary modelling, the current study has demonstrated the nutritional impact of meeting dairy recommendations using two types of commonly consumed milk products for children aged one to five years. Powdered milk can improve nutrient adequacy in calcium, phosphorus, sodium, vitamin A and riboflavin. YCM and PCM can bring additional nutritional benefits of a wider range of nutrients such as iron, vitamins C, D, E and folate, which are not typically found in other milks or foods commonly consumed by Filipino children.

Dairy consumption should be encouraged as part of the strategy to reduce nutrient inadequacies. Calcium, iron, folate, vitamins D and E are of concern in the Philippines as the level of inadequacies are extremely high in early years. Natural foods without fortification or supplements are unlikely to be sufficient to eliminate these inadequacies in the Philippines. Given that nutritional compositions vary amongst different milk products, caregivers and health care professionals should therefore encourage consumption of milk products that are fortified with key nutrients to ensure that children’s needs are met.

## Figures and Tables

**Table 1:** Sample population characteristics.

		Children Not Meeting Dairy Recommendations (*n* = 2642)		Children Meeting Dairy Recommendations (*n* = 1222)		All (*n* = 3864)	Chi-Square Test * p-Value
		*n*	%	*n*	%	*n*	
**Gender**	Female	1336	70	582	30	1918	<0.001
	Male	1306	67	640	33	1946	
**Age group**	1 to 2 years	792	54	669	46	1461	<0.001
	3 to 4 years	1136	74	395	26	1531	
	5 years	714	82	158	18	872	
**Dwelling location**	Urban	1662	77	505	23	2167	<0.001
	Rural	980	58	717	42	1697	
**Wealth quintiles**	Poorest	495	67	245	33	740	<0.001
	Poor	614	78	174	22	788	
	Middle	990	87	143	13	1133	
	Rich	302	49	319	51	621	
	Richest	168	35	311	65	479	
**BMI z-scores**	<−2 (Wasting)	155	67	77	33	232	<0.001
	−2 to −1	598	76	191	24	789	
	−1 to 0	1078	73	397	27	1475	
	0 to 1	573	64	318	36	891	
	1 to 2 (At risk of overweight)	140	53	124	47	264	
	>2 (Overweight)	98	46	115	54	213	

* chi-square tests were performed to test the relationships between each categorical variable (e.g., gender, age group, dwelling location, wealth quintiles, and BMI z-scores) and the binary variable of meeting dairy recommendations or not. BMI= Body Mass Index.

**Table 2:** Mean nutrient intakes and inadequacy at baseline and after modelling scenarios 1 and 2, in children aged one to two years (*n* = 792).

	Reference Intake	Baseline			Scenario 1 After Adding 1 Serving of Milk			Scenario 2 After Adding One Serving of YCM			*p*-Value *
	M/F	Mean	SD	% Children below Reference Intake ^1,2,3^	Mean	SD	% Children below Reference Intake ^1,2,3^	Mean	SD	% Children below Reference Intake ^1,2,3^	
Energy (kcal)	1000/920 ^1^	555.9	337.3	89	692.3	337.3	82	680.6	337.3	83	<0.001
Protein (g)	15/14	16.5	11.4	52	23.5	11.4	20	20.3	11.4	36	<0.001
Total fat (g)	25–35%E	11.0	10.0	91	18.5	10.0	82	16.4	10.0	85	<0.001
Saturated fat (g)	-	4.8	8.4	-	6.51	8.43	-	6.1	8.4	-	<0.001
Monounsaturated (g)	-	3.4	4.1	-	7.82	4.13	-	5.8	4.1	-	<0.001
Polyunsaturated (g)	-	1.7	2.2	-	2.81	2.16	-	2.8	2.2	-	<0.001
Carbohydrates (g)	50–69%E	97.6	60.8	5	107.7	60.8	14	112.8	60.8	6	<0.001
Fibre (g)	6–7	3.0	2.5	92	3.0	2.5	92	3.8	2.5	88	<0.001
Total sugars (g)	-	15.8	18.3	-	25.93	18.28	-	31.0	18.3	-	<0.001
Sodium (mg)	225 ^2^	429.0	503.9	41	554.1	503.9	21	499.6	503.9	30	<0.001
Calcium (mg)	440	128.1	105.2	99	356.7	105.2	83	301.4	105.2	91	<0.001
Phosphorus (mg)	380	255.9	164.4	80	441.3	164.4	42	363.3	164.4	62	<0.001
Iron (mg)	6.4/7.0	3.0	2.6	93	3.1	2.6	93	4.9	2.6	75	<0.001
Magnesium (mg)	60/60 ^2^	55.1	44.5	65	55.1	44.5	65	70.4	44.5	50	<0.001
Potassium (mg)	1000 ^2^	375.0	281.2	97	415.1	281.2	96	592.9	281.2	90	<0.001
Selenium (μg)	13.6/13.0	30.2	23.5	23	31.2	23.5	20	35.5	23.5	10	<0.001
Zinc (mg)	2.8/2.6	1.9	1.8	78	2.3	1.8	70	3.2	1.8	46	<0.001
Vitamin A	193/180	147.4	225.3	72	350.8	225.3	0	284.4	225.3	33	<0.001
Thiamin (mg)	0.4/0.4	0.3	0.3	74	0.4	0.3	67	0.6	0.3	29	<0.001
Riboflavin (mg)	0.4/0.4	0.3	0.2	78	0.7	0.2	0	0.5	0.2	31	<0.001
Niacin (mg)	5/5	5.1	3.8	57	5.3	3.8	56	7.3	3.8	33	<0.001
Vitamin B6 (mg)	0.4/0.5	0.4	0.3	68	0.4	0.3	68	0.7	0.3	18	<0.001
Vitamin B12 (mg)	0.8/0.9	1.0	1.6	61	1.0	1.6	61	1.6	1.6	38	<0.001
Folate (μg)	120/120	79.6	76.4	82	79.6	76.4	82	160.2	76.4	47	<0.001
Vitamin C (mg)	12/11	11.5	25.5	73	15.3	25.5	63	38.4	25.5	0	<0.001
Vitamin D (μg)	5 ^3^	1.0	2.0	98	1.0	2.0	98	2.9	2.0	93	<0.001
Vitamin E (mg)	4 ^3^	1.1	1.2	96	1.1	1.2	96	3.6	1.2	76	<0.001

* *p*-values represent significant differences in nutrient intakes between scenario 1 and scenario 2 using Wilcoxon signed-rank test. No reference values established for saturated fat, mono- and polyunsaturated fats, and total sugars. M = Male; F = Female. SD = Standard Deviation. YCM = Young Children Milk. %E = Percentage energy from fat or carbohydrates. ^1^ Recommended Energy Intake (REI); ^2^ Recommended Nutrient Intakes (RNI); ^3^ Adequate Intake (AI); Reference intakes w/o superscripts = Estimated Average Requirement.

**Table 3:** Mean nutrient intakes and inadequacy at baseline and after modelling scenarios 1 and 2, in children aged three to four years (*n* = 1136).

	Reference Intake	Baseline			Scenario 1 After Adding 1 Serving of Milk			Scenario 2 After Adding One Serving of PCM			*p*-Value *
	M/F	Mean	SD	% Children below Reference Intake ^1,2,3^	Mean	SD	% Children below Reference Intake ^1,2,3^	Mean	SD	% Children below Reference Intake ^1,2,3^	
Energy (kcal)	1350/1260 ^1^	853.3	379.4	88	989.8	379.4	81	970.3	379.4	82	<0.001
Protein (g)	18/17	25.7	13.0	29	32.8	13.0	7	29.9	13.0	14	<0.001
Total fat (g)	15–30%E	16.5	13.6	56	24.1	13.6	26	21.2	13.6	38	<0.001
Saturated fat (g)	-	7.3	9.7	-	8.94	9.72	-	8.6	9.7	-	<0.001
Monounsaturated (g)	-	5.4	7.2	-	9.81	7.15	-	7.5	7.2	-	<0.001
Polyunsaturated (g)	-	2.8	4.0	-	3.95	3.97	-	3.8	4.0	-	<0.001
Carbohydrates (g)	55–79%E	150.5	67.8	7	160.7	67.8	14	165.0	67.8	8	<0.001
Fibre (g)	8–10	4.6	2.7	93	4.6	2.7	93	5.5	2.7	90	<0.001
Total sugars (g)	-	21.8	22.0	-	31.94	22.03	-	36.3	22.0	-	<0.001
Sodium (mg)	300 ^2^	618.0	561.9	32	743.1	561.9	19	712.3	561.9	22	<0.001
Calcium (mg)	440	191.9	153.6	96	420.5	153.6	67	385.4	153.6	77	<0.001
Phosphorus (mg)	405	395.2	183.4	59	580.6	183.4	16	514.8	183.4	31	<0.001
Iron (mg)	7.5/7.4	4.8	3.5	75	4.9	3.5	75	6.5	3.5	55	<0.001
Magnesium (mg)	70/70 ^2^	87.0	57.9	43	87.0	57.9	43	105.0	57.9	24	<0.001
Potassium (mg)	1400 ^2^	587.9	362.8	97	628.0	362.8	96	802.8	362.8	94	<0.001
Selenium (μg)	16.1/15.6	45.9	26.4	8	46.9	26.4	7	50.8	26.4	3	<0.001
Zinc (mg)	3.3/3.2	2.9	1.9	67	3.3	1.9	59	4.3	1.9	32	<0.001
Vitamin A	226/214	234.8	438.6	64	438.2	438.6	7	336.9	438.6	37	<0.001
Thiamin (mg)	0.5/0.4	0.5	0.3	60	0.5	0.3	51	0.7	0.3	26	<0.001
Riboflavin (mg)	0.5/0.4	0.5	2.1	66	0.9	2.1	0	0.7	2.1	23	<0.001
Niacin (mg)	5/5	8.1	4.8	26	8.3	4.8	25	10.3	4.8	7	<0.001
Vitamin B6 (mg)	0.5/0.5	0.6	0.3	50	0.6	0.3	50	0.8	0.3	13	<0.001
Vitamin B12 (mg)	0.9/1.0	1.8	2.9	45	1.8	2.9	45	2.4	2.9	19	<0.001
Folate (μg)	160/160	120.4	101.6	76	120.4	101.6	76	180.9	101.6	53	<0.001
Vitamin C (mg)	17/17	17.0	29.2	73	20.7	29.2	67	35.8	29.2	0	<0.001
Vitamin D (μg)	5 ^3^	1.5	2.1	95	1.5	2.1	95	3.0	2.1	90	<0.001
Vitamin E (mg)	5 ^3^	1.8	1.8	96	1.8	1.8	96	3.5	1.7	88	<0.001

* *p*-values represent significant differences in nutrient intakes between scenario 1 and scenario 2 using Wilcoxon signed-rank test. No reference values established for saturated fat, mono- and polyunsaturated fats, and total sugars. M = Male; F = Female. SD = Standard Deviation. PCM = Preschool Children Milk. %E = Percentage energy from fat or carbohydrates. ^1^ Recommended Energy Intake (REI); ^2^ Recommended Nutrient Intakes (RNI); ^3^ Adequate Intake (AI); Reference intakes w/o superscripts = Estimated Average Requirement.

**Table 4:** Mean nutrient intakes and inadequacy at baseline and after modelling scenarios 1 and 2, in children aged five years (*n* = 714).

	Reference Intake	Baseline			Scenario 1 After Adding 1 Serving of Milk			Scenario 2 After Adding One Serving of PCM			*p*-Value *
	M/F	Mean	SD	% Children below Reference Intake ^1,2,3^	Mean	SD	% Children below Reference Intake ^1,2,3^	Mean	SD	% Children below Reference Intake ^1,2,3^	
Energy (kcal)	1350/1260 ^1^	950.5	381.6	83	1086.9	381.6	75	1062.6	381.6	76	<0.001
Protein (g)	18/17	29.3	13.2	18	36.4	13.2	3	33.9	13.2	7	<0.001
Total fat (g)	15–30%E	18.4	14.2	51	25.9	14.2	21	22.0	14.2	39	<0.001
Saturated fat (g)	-	8.1	10.6	-	9.80	10.63	-	9.4	10.6	-	<0.001
Monounsaturated (g)	-	5.5	5.5	-	9.88	5.47	-	7.0	5.5	-	<0.001
Polyunsaturated (g)	-	2.8	2.5	-	3.90	2.51	-	3.4	2.5	-	<0.001
Carbohydrates (g)	55–79%E	167.0	67.1	7	177.2	67.1	12	182.3	67.1	7	<0.001
Fibre (g)	8–10	5.1	2.8	92	5.1	2.8	92	5.9	2.8	89	<0.001
Total sugars (g)	-	22.2	19.5	-	32.35	19.48	-	37.0	19.5	-	<0.001
Sodium (mg)	300 ^2^	652.2	577.5	30	777.3	577.5	18	727.4	577.5	23	<0.001
Calcium (mg)	440	201.5	122.8	95	430.1	122.8	64	403.0	122.8	70	<0.001
Phosphorus (mg)	405	447.3	189.1	49	632.7	189.1	8	576.2	189.1	18	<0.001
Iron (mg)	7.5/7.4	5.3	5.6	85	5.4	5.6	85	7.1	5.6	65	<0.001
Magnesium (mg)	70/70 ^2^	92.9	43.6	33	92.9	43.6	33	111.7	43.6	13	<0.001
Potassium (mg)	1400 ^2^	646.0	364.6	96	686.1	364.6	95	941.5	364.6	92	<0.001
Selenium (μg)	16.1/15.6	51.7	27.4	4	52.8	27.4	4	57.1	27.4	1	<0.001
Zinc (mg)	3.3/3.2	3.2	2.0	61	3.6	2.0	51	4.6	2.0	24	<0.001
Vitamin A	226/214	248.1	414.7	64	451.5	414.7	4	352.9	414.7	32	<0.001
Thiamin (mg)	0.5/0.4	0.5	0.3	54	0.6	0.3	46	0.7	0.3	20	<0.001
Riboflavin (mg)	0.5/0.4	0.4	0.3	64	0.9	0.3	0	0.7	0.3	9	<0.001
Niacin (mg)	5/5	9.0	4.3	16	9.2	4.3	16	11.2	4.3	4	<0.001
Vitamin B6 (mg)	0.5/0.5	0.7	0.3	39	0.7	0.3	39	0.9	0.3	6	<0.001
Vitamin B12 (mg)	0.9/1.0	2.0	2.3	37	2.0	2.3	37	2.5	2.3	17	<0.001
Folate (μg)	160/160	125.1	101.2	74	125.1	101.2	74	185.5	101.2	50	<0.001
Vitamin C (mg)	17/17	17.3	29.9	72	21.0	29.9	65	37.4	29.9	0	<0.001
Vitamin D (μg)	5 ^3^	1.9	2.7	93	1.9	2.7	93	3.4	2.7	87	<0.001
Vitamin E (mg)	5 ^3^	1.8	1.4	95	1.8	1.4	95	3.9	1.4	83	<0.001

* *p*-values represent significant differences in nutrient intakes between scenario 1 and scenario 2 using Wilcoxon signed-rank test. No reference values established for saturated fat, mono- and polyunsaturated fats, and total sugars. M = Male; F = Female. SD = Standard Deviation. PCM = Preschool Children Milk. %E = Percentage energy from fat or carbohydrates. ^1^ Recommended Energy Intake (REI); ^2^ Recommended Nutrient Intakes (RNI); ^3^ Adequate Intake (AI); Reference intakes w/o superscripts = Estimated Average Requirement.

**Table 5:** Percentage of children aged one to two years with inadequate intakes.

	No/Low Dairy Consumers (*n* = 792)			All Children (*n* = 1461)		
	Baseline	Scenario 1	Scenario 2	Baseline	Scenario 1	Scenario 2
Energy (kcal)	89%	82%	83%	79%	75%	75%
Protein (g)	52%	20%	36%	34%	17%	26%
Total fat (g)	91%	82%	85%	72%	67%	69%
Carbohydrates (g)	5%	14%	6%	13%	18%	14%
Fibre (g)	92%	92%	88%	93%	93%	91%
Sodium (mg)	41%	21%	30%	29%	18%	23%
Calcium (mg)	99%	83%	91%	71%	62%	67%
Phosphorus (mg)	80%	42%	62%	59%	38%	49%
Iron (mg)	93%	93%	75%	75%	75%	65%
Magnesium (mg)	65%	65%	50%	62%	62%	54%
Potassium (mg)	97%	96%	90%	90%	89%	86%
Selenium (μg)	23%	20%	10%	22%	20%	15%
Zinc (mg)	78%	70%	46%	60%	56%	43%
Vitamin A	72%	0%	33%	60%	20%	38%
Thiamin (mg)	74%	67%	29%	60%	56%	35%
Riboflavin (mg)	78%	0%	31%	48%	5%	22%
Niacin (mg)	57%	56%	33%	51%	50%	38%
Vitamin B6 (mg)	68%	68%	18%	62%	62%	35%
Vitamin B12 (mg)	61%	61%	38%	55%	55%	42%
Folate (μg)	82%	82%	47%	75%	75%	56%
Vitamin C (mg)	73%	63%	0%	56%	51%	16%
Vitamin D (μg)	98%	98%	93%	87%	87%	85%
Vitamin E (mg)	96%	96%	76%	87%	87%	76%

**Table 6:** Percentage of children aged three to four years with inadequate intakes.

	No/Low Dairy Consumers (*n* = 1136)			All Children (*n* = 1531)		
	Baseline	Scenario 1	Scenario 2	Baseline	Scenario 1	Scenario 2
Energy (kcal)	88%	81%	82%	84%	79%	80%
Protein (g)	29%	7%	14%	22%	7%	12%
Total fat (g)	56%	26%	38%	45%	22%	31%
Carbohydrates (g)	7%	14%	8%	14%	19%	15%
Fibre (g)	93%	93%	90%	94%	94%	91%
Sodium (mg)	32%	19%	22%	26%	17%	19%
Calcium (mg)	96%	67%	77%	84%	62%	69%
Phosphorus (mg)	59%	16%	31%	48%	16%	27%
Iron (mg)	75%	75%	55%	75%	75%	55%
Magnesium (mg)	43%	43%	24%	44%	44%	29%
Potassium (mg)	97%	96%	94%	95%	95%	94%
Selenium (μg)	8%	7%	3%	8%	7%	4%
Zinc (mg)	67%	59%	32%	60%	53%	34%
Vitamin A	64%	7%	37%	56%	13%	36%
Thiamin (mg)	60%	51%	26%	54%	47%	29%
Riboflavin (mg)	66%	0%	23%	52%	3%	19%
Niacin (mg)	26%	25%	7%	25%	23%	11%
Vitamin B6 (mg)	50%	50%	13%	48%	48%	21%
Vitamin B12 (mg)	45%	45%	19%	44%	44%	25%
Folate (μg)	76%	76%	53%	73%	73%	56%
Vitamin C (mg)	73%	67%	0%	68%	64%	14%
Vitamin D (μg)	95%	95%	90%	93%	93%	90%
Vitamin E (mg)	96%	96%	88%	94%	94%	87%

**Table 7:** Percentage of children aged five years with inadequate intakes.

	No/Low Dairy Consumers (*n* = 714)			All Children (*n* = 872)		
	Baseline	Scenario 1	Scenario 2	Baseline	Scenario 1	Scenario 2
Energy (kcal)	83%	75%	76%	78%	72%	73%
Protein (g)	18%	3%	7%	15%	3%	6%
Total fat (g)	51%	21%	39%	43%	18%	32%
Carbohydrates (g)	7%	12%	7%	10%	14%	10%
Fibre (g)	92%	92%	89%	93%	93%	90%
Sodium (mg)	30%	18%	23%	25%	15%	19%
Calcium (mg)	95%	64%	70%	88%	62%	67%
Phosphorus (mg)	49%	8%	18%	42%	9%	17%
Iron (mg)	85%	85%	65%	85%	85%	65%
Magnesium (mg)	33%	33%	13%	33%	33%	16%
Potassium (mg)	96%	95%	92%	95%	95%	92%
Selenium (μg)	4%	4%	1%	4%	3%	1%
Zinc (mg)	61%	51%	24%	56%	48%	26%
Vitamin A	64%	4%	32%	57%	8%	31%
Thiamin (mg)	54%	46%	20%	50%	43%	23%
Riboflavin (mg)	64%	0%	9%	53%	1%	8%
Niacin (mg)	16%	16%	4%	15%	15%	6%
Vitamin B6 (mg)	39%	39%	6%	37%	37%	10%
Vitamin B12 (mg)	37%	37%	17%	36%	36%	19%
Folate (μg)	74%	74%	50%	71%	71%	52%
Vitamin C (mg)	72%	65%	0%	69%	63%	10%
Vitamin D (μg)	93%	93%	87%	93%	93%	88%
Vitamin E (mg)	95%	95%	83%	95%	95%	84%

## Supplementary Materials

The following are available online at https://www.mdpi.com/2072-6643/12/11/3330/s1, Table S1. Compositions of one serving (180 g) of the milk products used in the modelling scenarios. Table S2. Mean daily vitamin A intakes, percentages below EAR and above Upper Limit, based on potential impact of vitamin A (µg RE/day) supplementation in children one to five years.
