# Supplementary material for: The Nutritional Impact of Milk Beverages in Reducing Nutrient Inadequacy among Children Aged One to Five Years in the Philippines: A Dietary Modelling Study
Source: Nutrients. 2020 Oct 29;12(11):3330. doi: 10.3390/nu12113330 (PMC7692769; doi:10.3390/nu12113330)
Supplement: Supplementary file 1 [file nutrients-12-03330-s001.pdf]

Supplementary information

Table S1. Compositions of one serving (180g) of the milk beverages used in the modelling scenarios

	Powdered Milk	YCM (1-2 years)	PCM (3-4 years)	PCM (5 years)
Energy (kcal)	136.4	124.66	117	112.16
Protein (g)	7.0	3.87	4.16	4.57
Total fat (g)	7.5	5.37	4.7	3.63
Saturated fats (g)	1.7	1.3	2.1	1.2
Monounsaturated fats (g)	4.4	2.4	2.1	1.5
Polyunsaturated fats (g)	1.1	1.1	1.0	0.7
Carbohydrates (g)	10.2	15.21	14.51	15.31
Fibre (g)	0.0	0.81	0.81	0.81
Total sugars (g)	10.2	15.2	14.5	14.8
Sodium (mg)	125.1	70.66	94.3	75.22
Calcium (mg)	228.6	173.28	193.43	201.49
Phosphorus (mg)	185.4	107.46	119.55	128.96
Iron (mg)	0.1	1.88	1.75	1.75
Magnesium (mg)	0.0	15.31	18	18.81
Potassium (mg)	40.1	217.88	214.93	295.52
Selenium (μg)	1.1	5.37	4.97	5.37
Zinc (mg)	0.4	1.32	1.4	1.34
Vitamin A	203.4	137.01	102.09	104.78
Thiamin (mg)	0.1	0.27	0.21	0.21
Riboflavin (mg)	0.5	0.27	0.26	0.31
Niacin (mg)	0.2	2.15	2.2	2.2
Vitamin B6 (mg)	0.0	0.32	0.24	0.25
Vitamin B12 (mg)	0.0	0.54	0.62	0.54
Folate (μg)	0.0	80.6	60.45	60.45
Vitamin C (mg)	3.8	26.87	18.81	20.15
Vitamin D (μg)	0.0	1.88	1.45	1.45
Vitamin E (mg)	0.0	2.45	1.77	2.13

Table S2. Mean daily vitamin A intakes, percentages below EAR and above Upper Limit, based on potential impact of vitamin A (μg RE/day) supplementation in children one to five years

	1 to 2 years	3 to 4 years	5 years

Daily intake from supplementation* only	330	330	330
% children below EAR	0	0	0
Daily intake (±SD) from supplementation* + baseline diet	477,4 (±225,3)	564,8 (±438,6)	578,1 (±414,7)
% children below EAR	0	0	0
% children above UL	2	3	2
Daily intake (±SD) from supplementation* + Scenario 1	680,8 (±225,3)	768,2 (±438,6)	781,5 (±414,7)
% children below EAR	0	0	0
% children above UL	61	21	15
Daily intake (±SD) from supplementation* + Scenario 2	614,4 (±225,3)	666,9 (±438,6)	682,9 (±414,7)
% children below EAR	0	0	0
% children above UL	39	12	8

*Assuming all children received supplementation
